# New England

**Published:** 1899-06

**Authors:** A. J. Sheldon

**Affiliations:** Secretary


Secretary.

### NEW ENGLAND ASSOCIATION OF THE AMERICAN VETERINARY COLLEGE, STATE OF MASSACHUSETTS.

A meeting to organize the New England Alumni Association of the American Veterinary College was called at Boston, Mass., April 19th. The following members of the profession, graduates of this college, were present: Drs. George H. Bailey, F. W. Huntington, George F. Westcott, of Portland, Maine; W. L. La Baw, L. H. Howard, Austin Peters, and A. J. Sheldon, Boston; G. Bickell, Haverhill; W. H. Dodge, Leominster; J. J. Riordan, Beverly Farms; C. H. Tilton, Ashland; J. J. Monahan, Holyoke, Mass.; George Stephens, White River Junction, Vt.

Dr. L. H. Howard was appointed temporary chairman, and Dr. Austin Peters, Secretary. Dr. J. F. Winchester proposed that the New England Alumni form a permanent organization; carried.

Officers were elected as follows: President, J. F. Winchester; Secretary-Treasurer, A. J. Sheldon; Executive Committee, J. F. Winchester, A. J. Sheldon (ex-officio), George H. Bailey, W. H. Dodge, and C. L. Adams.

It was voted to incorporate under the name of the New England Alumni Association of the American Veterinary College (State of Massachusetts).

Voted that each member attending the silver anniversary of the American Veterinary College be considered an official delegate of the Association.

At 9.30 P.M. the meeting adjourned to a banquet for about two hours, concluding with happy toasts.

The next annual meeting will be held in Boston, April, 1900.

A. J. SHELDON,
Secretary.

